# Supplementary material for: Causal Relationship between Aging and Anorexia Nervosa: A White-Matter-Microstructure-Mediated Mendelian Randomization Analysis
Source: Biomedicines. 2024 Aug 16;12(8):1874. doi: 10.3390/biomedicines12081874 (PMC11351342; doi:10.3390/biomedicines12081874)
Supplement: Supplementary file 1 [file biomedicines-12-01874-s001.zip › Supplementary Figure/Figure S4/Singe SNP plot of MR analysis for TL to ubm-b-1870.pdf]

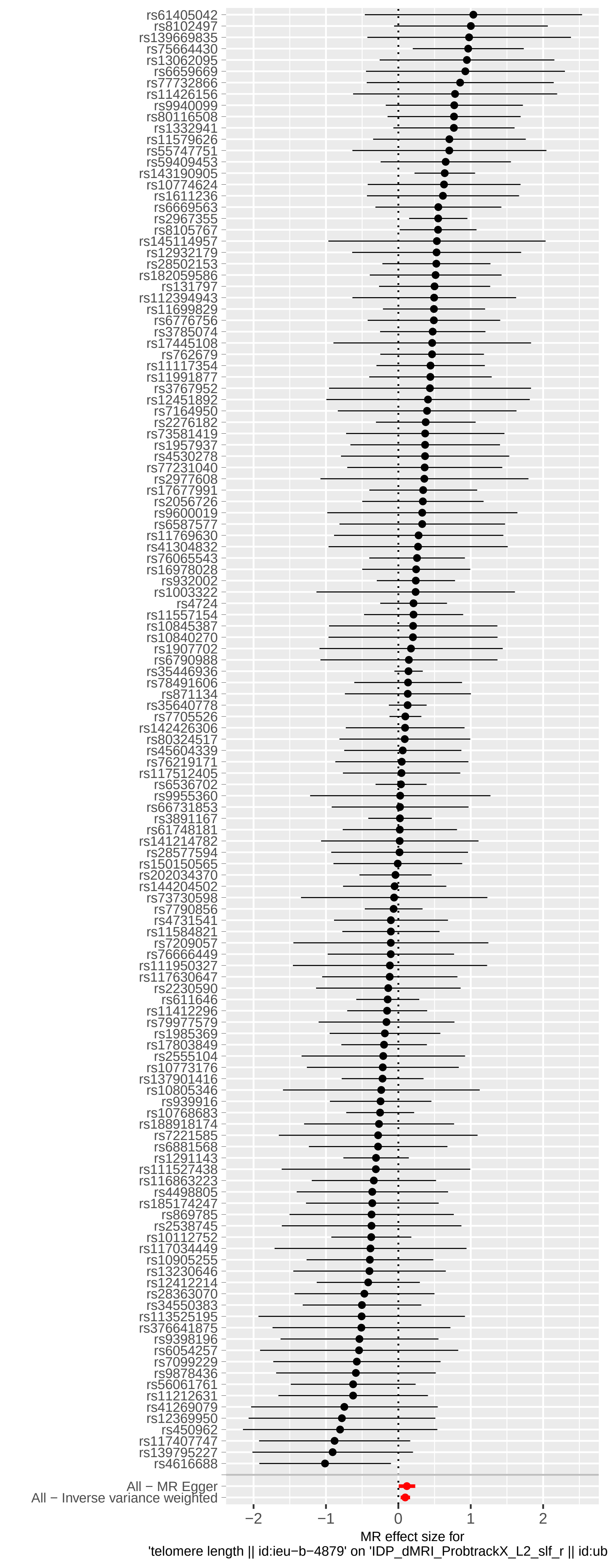
rs61405042
rs8102497
rs139669835
rs75664430
rs13062095
rs6659669
rs77732866
rs11426156
rs9940099
rs80116508
rs1332941
rs11579626
rs55747751
rs59409453
rs143190905
rs10774624
rs1611236
rs6669563
rs2967355
rs8105767
rs145114957
rs12932179
rs28502153
rs182059586
rs131797
rs112394943
rs11699829
rs6776756
rs3785074
rs17445108
rs762679
rs11117354
rs11991877
rs3767952
rs12451892
rs7164950
rs2276182
rs73581419
rs1957937
rs4530278
rs77231040
rs2977608
rs17677991
rs2056726
rs9600019
rs6587577
rs11769630
rs41304832
rs76065543
rs16978028
rs932002
rs1003322
rs4724
rs11557154
rs10845387
rs10840270
rs1907702
rs6790988
rs35446936
rs78491606
rs871134
rs35640778
rs7705526
rs142426306
rs80324517
rs45604339
rs76219171
rs117512405
rs6536702
rs9955360
rs66731853
rs3891167
rs61748181
rs141214782
rs28577594
rs150150565
rs202034370
rs144204502
rs73730598
rs7790856
rs4731541
rs11584821
rs7209057
rs76666449
rs111950327
rs117630647
rs2230590
rs611646
rs11412296
rs79977579
rs1985369
rs17803849
rs2555104
rs10773176
rs137901416
rs10805346
rs939916
rs10768683
rs188918174
rs7221585
rs6881568
rs1291143
rs111527438
rs116863223
rs4498805
rs185174247
rs869785
rs2538745
rs10112752
rs117034449
rs10905255
rs13230646
rs12412214
rs28363070
rs34550383
rs113525195
rs376641875
rs9398196
rs6054257
rs7099229
rs9878436
rs56061761
rs11212631
rs41269079
rs12369950
rs450962
rs117407747
rs139795227
rs4616688
All − MR Egger
All − Inverse variance weighted
−2
−1
0
1
2
MR effect size for
'telomere length || id:ieu−b−4879' on 'IDP_dMRI_ProbtrackX_L2_slf_r || id:ub
